# Supplementary material for: Early adversity promotes adolescent avoidance behavior by enhancing prefrontal–amygdala communication through CRH+ glutamatergic neurons
Source: bioRxiv. 2026 Sep 20:2025.12.23.696229. Preprint. [Version 2] doi: 10.64898/2025.12.23.696229 (PMC13596144; doi:10.64898/2025.12.23.696229)
Supplement: Supplementary file 1 [file media-1.pdf]

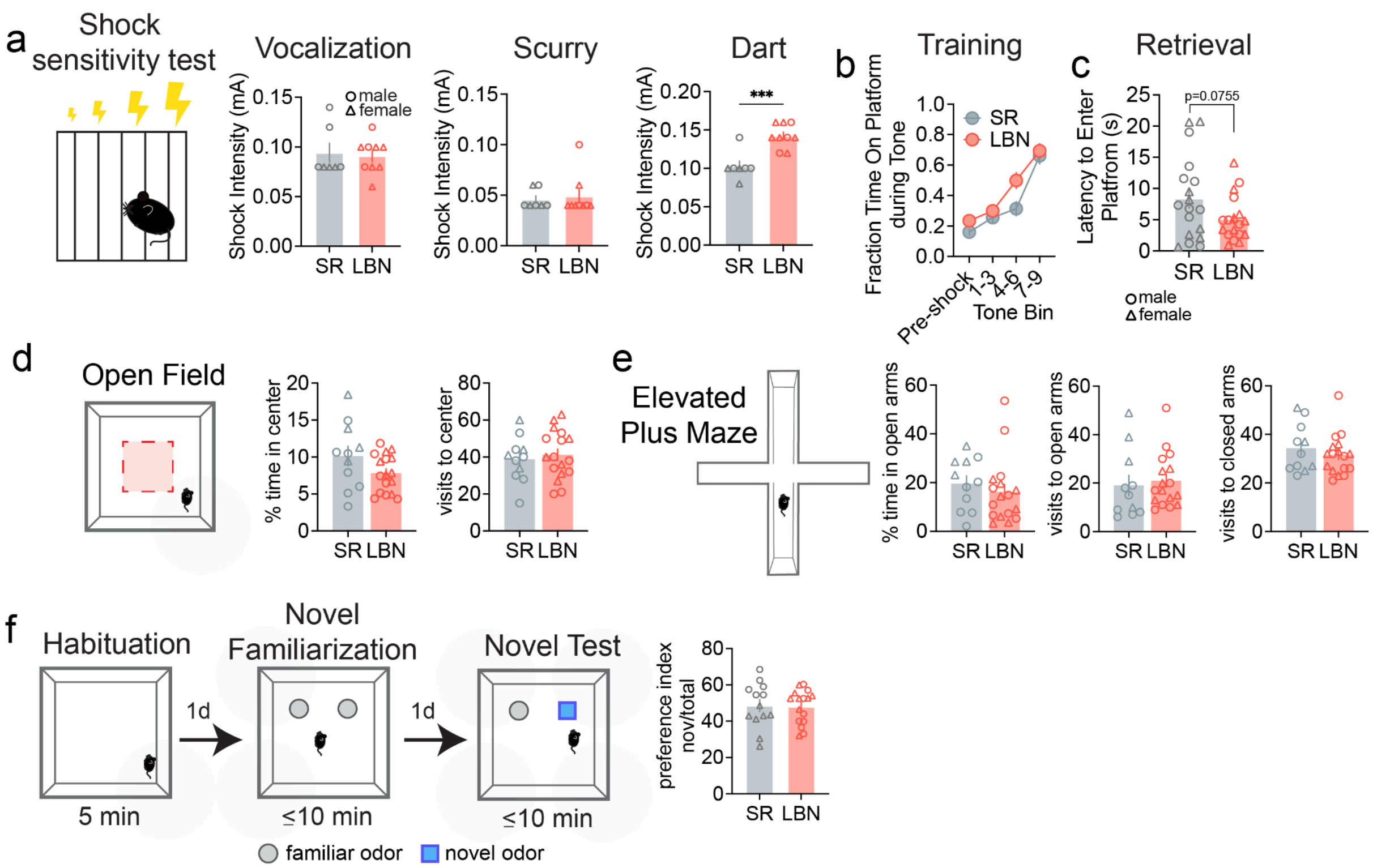

Extended Data Figure 1. Effects of LBN on shock sensitivity, open field, elevated plus maze and novel odor assay in adolescent mice, Related to Figure 1. (**a**) Summary data of shock levels required to elicit behavioral responses to progressive increases in shock intensity (SR, n=7; LBN, n=9 mice) (unpaired t-tests Vocalization $F_{6,8}$=2.02, p=0.770; Scurry: $F_{6,8}$=4.32, p=0.710; Dart: $F_{6,8}$=1.325, P=0.0004). (**b**) Fraction of time spent on the platform during PMA training (two-way repeated measures ANOVA with Sidak's post hoc test: trial effect $F_{2.48,86.71}$ = 69.69, P<0.0001; rearing effect $F_{1,35}$ = 4.79, P=0.036; interaction $F_{2.48,86.71}$= 1.92, P = 0.143). (**c**) Latency to enter the platform after tone onset in seconds (Mann-Whitney unpaired t-test $F_{17,17}$=3.38, p=0.0755). (**d**) Summary data of open field assay (SR, n=11; LBN, n=17 mice) including the time in center (unpaired t-test with Welch's correction $F_{10,16}$=3.110, p=0.1409), and visits to center (unpaired t-test $F_{16,10}$=1.146, p=0.637). (**e**) Summary data of elevated plus maze assay (SR, n=11; LBN, n=17) including time in the open arms (unpaired t-test $F_{16,10}$=1.579, p=0.487, visits to the open arms (unpaired t-test $F_{10,16}$=1.64, p=0.690) and visits to the closed arms (unpaired t-test $F_{10,16}$=1.373, p=0.450). (**f**) Summary data for novel odor assay (SR male, n=6; SR female, n=7, LBN male, n=8, LBN female, n=8 mice) measured as preference index (novel/total) (unpaired t-test $F_{12,15}$=1.74, p=0.889). Data are shown as mean +/- s.e.m. Triangles denote females, and circles denote males.

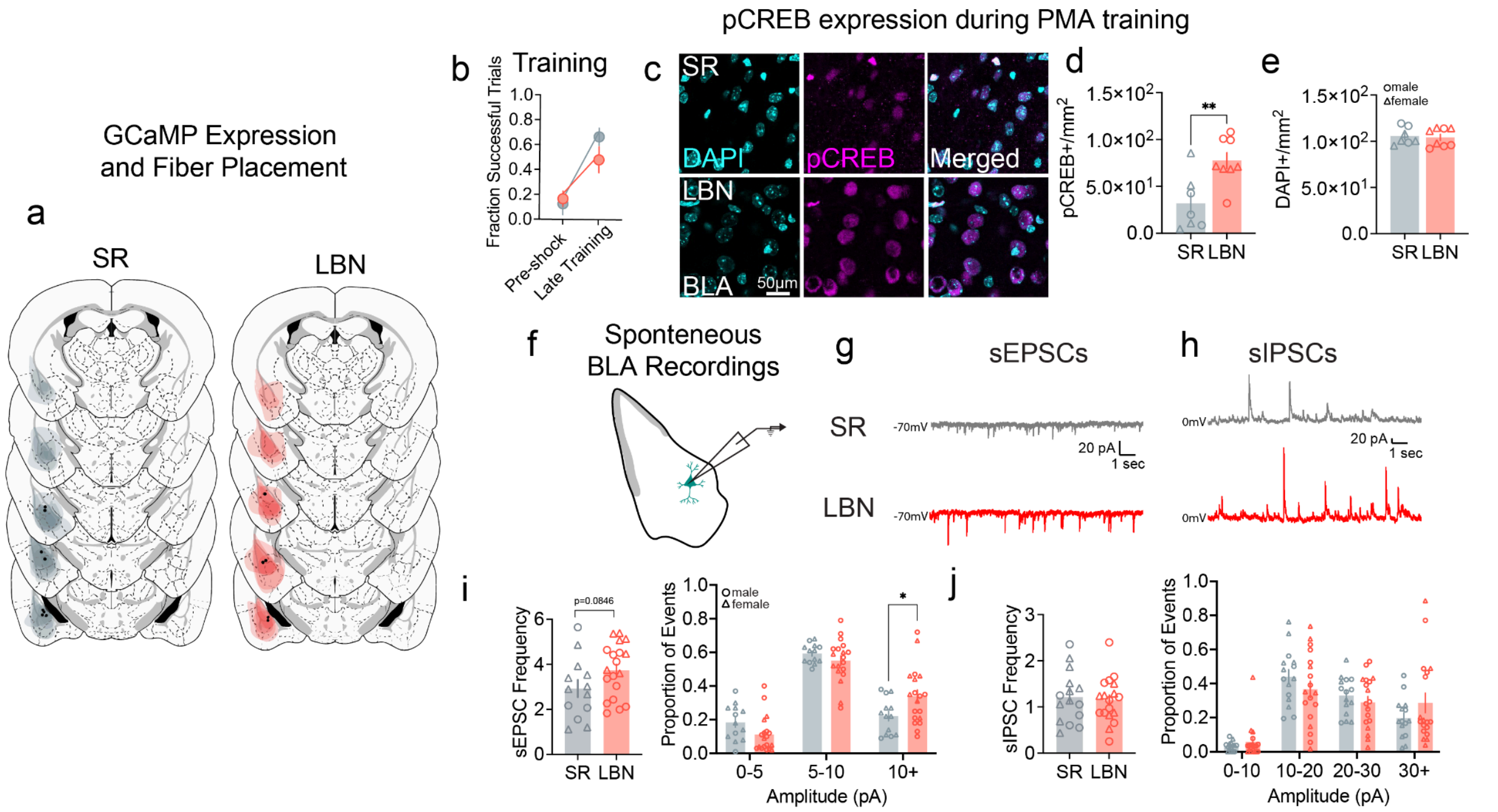

Extended Data Figure 2. LBN enhances excitability via pCREB expression and spontaneous vesicle release in the BLA, Related to Figure 1. (**a**) AAV-GCaMP6s viral expression and fiber implant placement in BLA for SR and LBN mice. (**b**) Fraction of successful trials during PMA training of animals taken for pCREB expression (SR, n=7; LBN, n=8 mice) (2-way ANOVA: trial effect $F_{1,29}$=25.25, p<0.0001; rearing effect $F_{1,29}$=0.71, p=0.407; interaction effect $F_{1,29}$=1.78, p=0.194). (**c**) Example of DAPI, pCREB and merged images taken at 63x in BLA following PMA training. (**d**) number of pCREB+ cells (unpaired t-test $F_{6,7}$=1.44, p=0.006) and (**e**) DAPI cells (unpaired t-test $F_{7,6}$=1.14, p=0.794) recorded per mm^2. (**f**) Schematic of whole cell patch clamp recordings of spontaneous postsynaptic currents. Representative traces of (**g**) sEPSCs and (**h**) sIPSCs in LBN and SR adolescent mice (EPSCs: SR, n=7 mice/13 cells; LBN, n=10 mice/19 cells; IPSCs: SR, n=7 mice/15 cells; LBN, n=10 mice/18 cells). Quantification of (**i**) sEPSC and (**j**) sIPSC frequency (sEPSC: unpaired t-test $F_{12,18}$=1.31, p=0.085; sIPSC: unpaired t-test $F_{14,17}$=1.32, p=0.737) and amplitudes (sEPSC: two-way ANOVA with Sidak's post hoc test: bin effect $F_{2,60}$=65.63, p<0.0001, rearing effect $F_{1,30}$=5.12, p=0.031, interaction effect $F_{2,60}$=3.61, p=0.033; sIPSC: two-way ANOVA with Sidak's post hoc test: bin effect $F_{3,93}$=22.14, p<0.0001, rearing effect $F_{1,31}$=0.087, p=0.770, interaction effect $F_{3,93}$=1,25, p=0.297). Data are shown as mean +/- s.e.m.; *P≤0.05, **P≤0.01. Triangles denote females, and circles denote males.

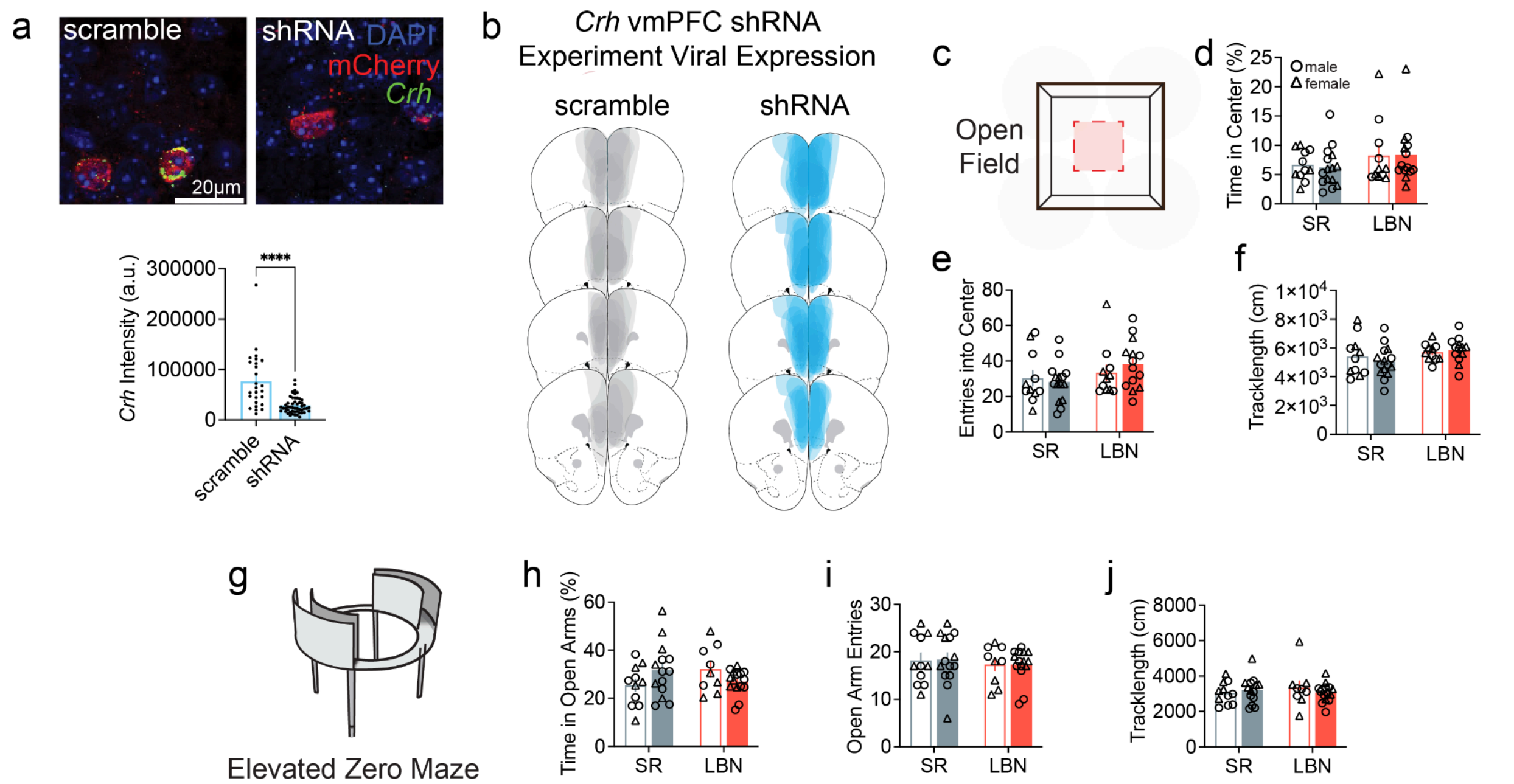

Extended Data Figure 3. Histology and anxiety-like behaviors for *Crh* shRNA knockdown in vmPFC experiment, Related to Figure 2. (**a**) Example images of in situ hybridization labelling Crh expression (green) in mCherry+ cells (red) in scramble controls versus shRNA knockdown mice. Quantification of Crh expression between conditions (Mann-Whitney test (two-tailed) p=0.0007; scramble, n=26 cells/2 mice; shRNA, n=51 cells/4 mice). (**b**) AAV-shRNA viral expression in mPFC of CRH-cre adolescent mice. (**c**) Summary data of open field assay (SR scramble, n=11; SR shRNA, n=15, LBN scramble, n=11; LBN shRNA, n=14 mice) including the (**d**) time in center (two-way ANOVA: interaction effect $F_{1,47}$=0.036, p=0.851, rearing effect $F_{1,47}$=2.36, p=0.133, knockdown effect $F_{1,47}$=0.013, p=0.910), (**e**) entries into the center (two-way ANOVA: interaction effect $F_{1,47}$=0.82, p=0.271, rearing effect $F_{1,47}$=3.06, p=0.087, knockdown effect $F_{1,47}$=0.11, p=0.737) and (**f**) tracklength in cm (two-way ANOVA: interaction effect $F_{1,47}$=0.85, p=0.362, rearing effect $F_{1,47}$=3.65 p=0.062, knockdown effect $F_{1,47}$=0.11, p=0.740). (**g**) Summary data of elevated plus maze assay (SR scramble, n=11; SR shRNA, n=14, LBN scramble, n=9; LBN shRNA, n=15 mice) including (**h**) time in the open arms (two-way ANOVA: interaction effect $F_{1,45}$=5.11, p=0.023, rearing effect $F_{1,45}$=0.135 p=0.715, rearing effect $F_{1,45}$=0.057, p=0.812), (**i**) visits to the open arms (two-way ANOVA: interaction effect $F_{1,45}$=0.0043, p=0.948, rearing effect $F_{1,45}$=0.49 p=0.488, knockdown effect $F_{1,45}$=0.0043, p=0.948) and (**j**) tracklength (two-way ANOVA: interaction effect $F_{1,45}$=1.0, p=0.323, rearing effect $F_{1,45}$=0.144 p=0.701, knockdown effect $F_{1,45}$=0.045 p=0.832. Data are shown as mean +/- s.e.m. Triangles denote females, and circles denote males.

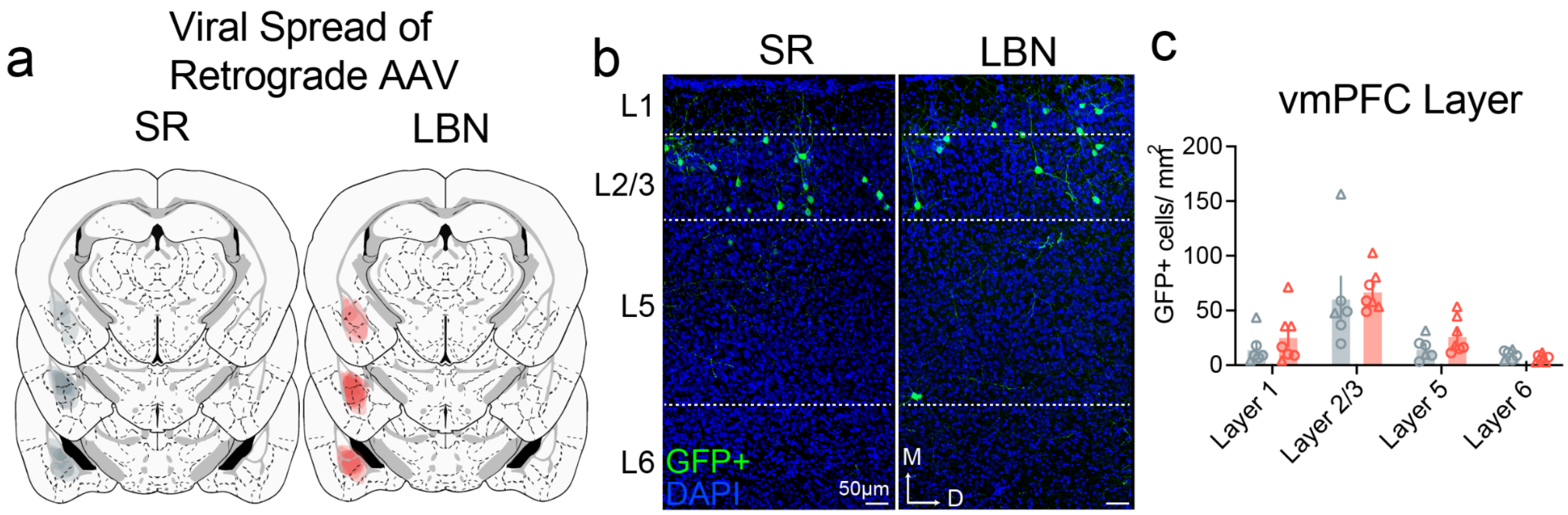

Extended Data Figure 4. Distribution of CRH+ cells that project to the BLA in throughout the brain and within the vmPFC, Related to Figure 3. (**a**) AAVrg-FLEX-GFP viral expression in BLA for SR and LBN animals. (**b**) Example images of CRH+ that project to the BLA in layers of the vmPFC (SR, n=6; LBN, n=7 mice). (**c**) Quantification of layer analysis (two-way repeated measures ANOVA with Sidak's post hoc test: layer effect $F_{1.48,16.31} = 19.41$, P=0.001; rearing effect $F_{1,11} = 0.99$, P=0.342; interaction $F_{1.48,16.31} = 0.35$, P = 0.647). All data points represent biological replicates. Data are shown as mean +/- s.e.m. Triangles denote females, and circles denote males.

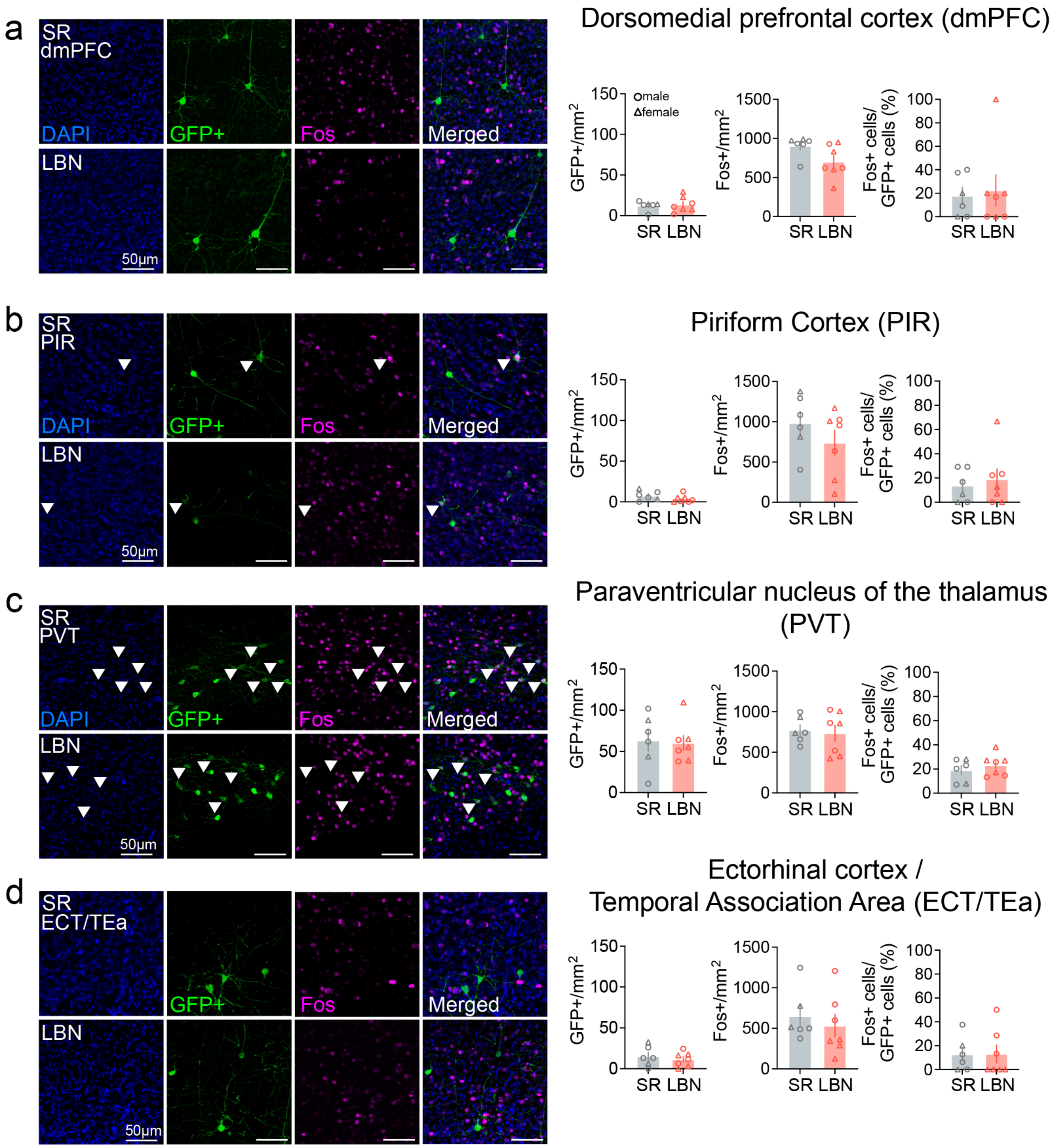

Extended Data Figure 5. Activation of retrogradely labelled regions following PMA training. Related to Figure 3. (**a**) Examples of DAPI (blue), retrogradely labelled CRH$^+$ cells (green), Fos+ cells (magenta) and merged images in the dmPFC. Quantification of the number of GFP$^+$ cells (unpaired t-test $F_{5,6}$=1.64, p=0.231), Fos+ cells (unpaired t-test $F_{6,5}$=2.52, p=0.070) and the percent of GFP$^+$/Fos+ cells (unpaired t-test $F_{6,5}$=2.96, p=0.779). (**b**) Examples of DAPI (blue), retrogradely labelled CRH$^+$ cells (green), Fos+ cells (magenta) and merged images in the piriform cortex (PIR). Quantification of the number of GFP$^+$ cells (unpaired t-test $F_{6,5}$=2.78, p=0.756), Fos+ cells (unpaired t-test $F_{6,5}$=1.35, p=0.280) and the percent of GFP$^+$/Fos+ cells (unpaired t-test $F_{6,5}$=2.93, p=0.637). (**c**) Examples of DAPI (blue), retrogradely labelled CRH$^+$ cells (green),

Fos+ cells (magenta) and merged images in the paraventricular nucleus of the thalamus (PVT). Quantification of the number of GFP^+ cells (unpaired t-test $F_{5,6}$=1.82, p=0.849), Fos+ cells (unpaired t-test $F_{6,5}$=2.61, p=0.762) and the percent of GFP^+/Fos+ cells (unpaired t-test $F_{5,6}$=1.14, p=0.418). (**d**) Examples of DAPI (blue), retrogradely labelled CRH^+ cells (green), Fos+ cells (magenta) and merged images in the ectorhinal cortex and temporal association area (ECT/TEa). Quantification of the number of GFP^+ cells (unpaired t-test $F_{5,6}$=1.94, p=0.525), Fos+ cells (unpaired t-test $F_{6,5}$=1.32, p=0.554) and the percent of GFP^+/Fos+ cells (unpaired t-test $F_{6,5}$=1.85, p=0.967). White arrows point to GFP+/Fos+ cells. All data points represent biological replicates. Data are shown as mean +/- s.e.m. Triangles denote females, and circles denote males.

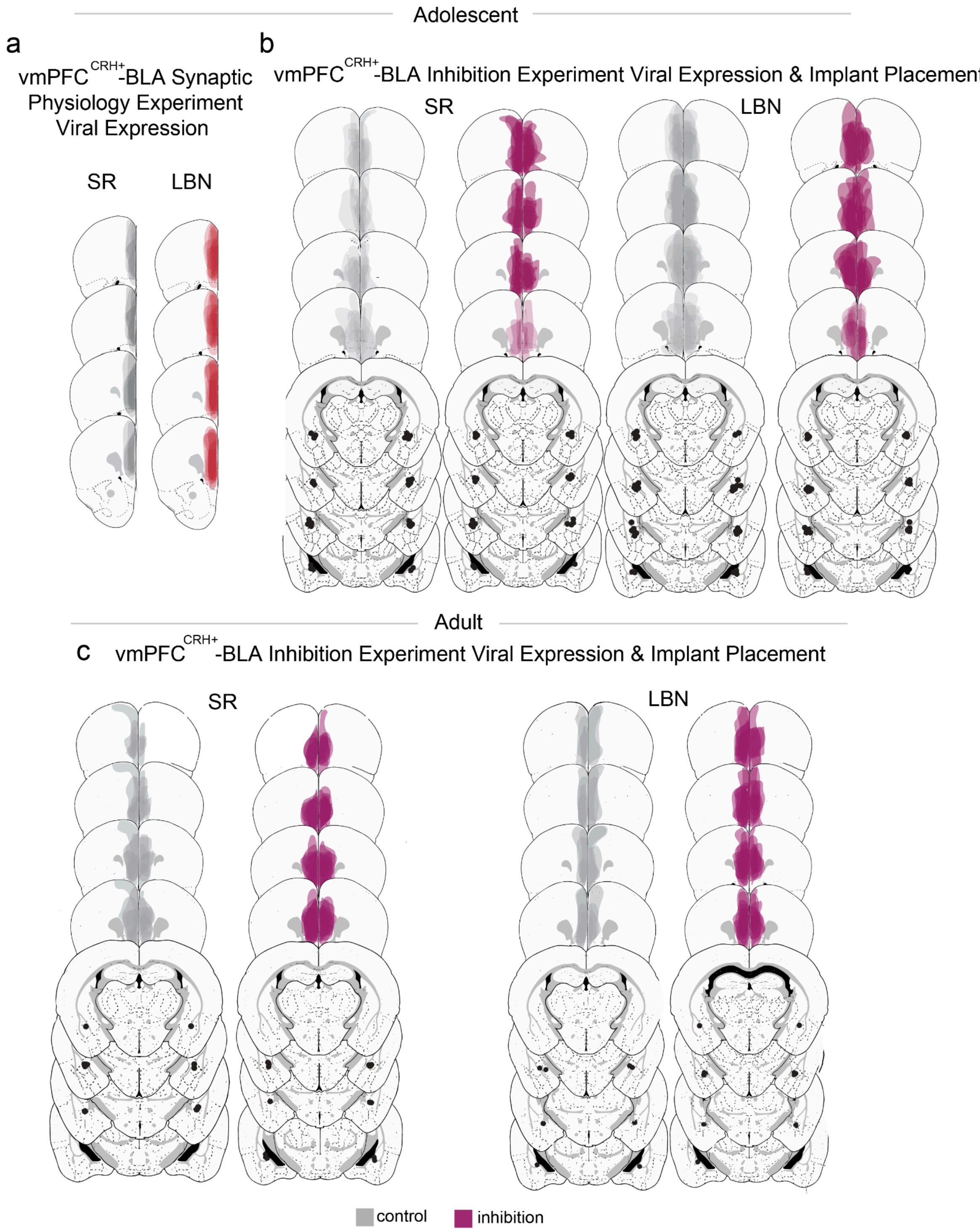

Extended Data Figure 6. Histology for vmPFC^{CRH+} → BLA synaptic physiology and optogenetic manipulation experiments, Related to Figures 3 and 4. (**a**) AAV-ChR2 viral expression in mPFC of SR and LBN mice for synaptic physiology experiments. (**b**) AAV-PdCO or control viral expression in mPFC of SR and LBN adolescent mice and fiber implant placement above BLA. (**c**) AAV-PdCO or control viral expression in mPFC of SR and LBN adult mice and fiber implant placement above BLA.

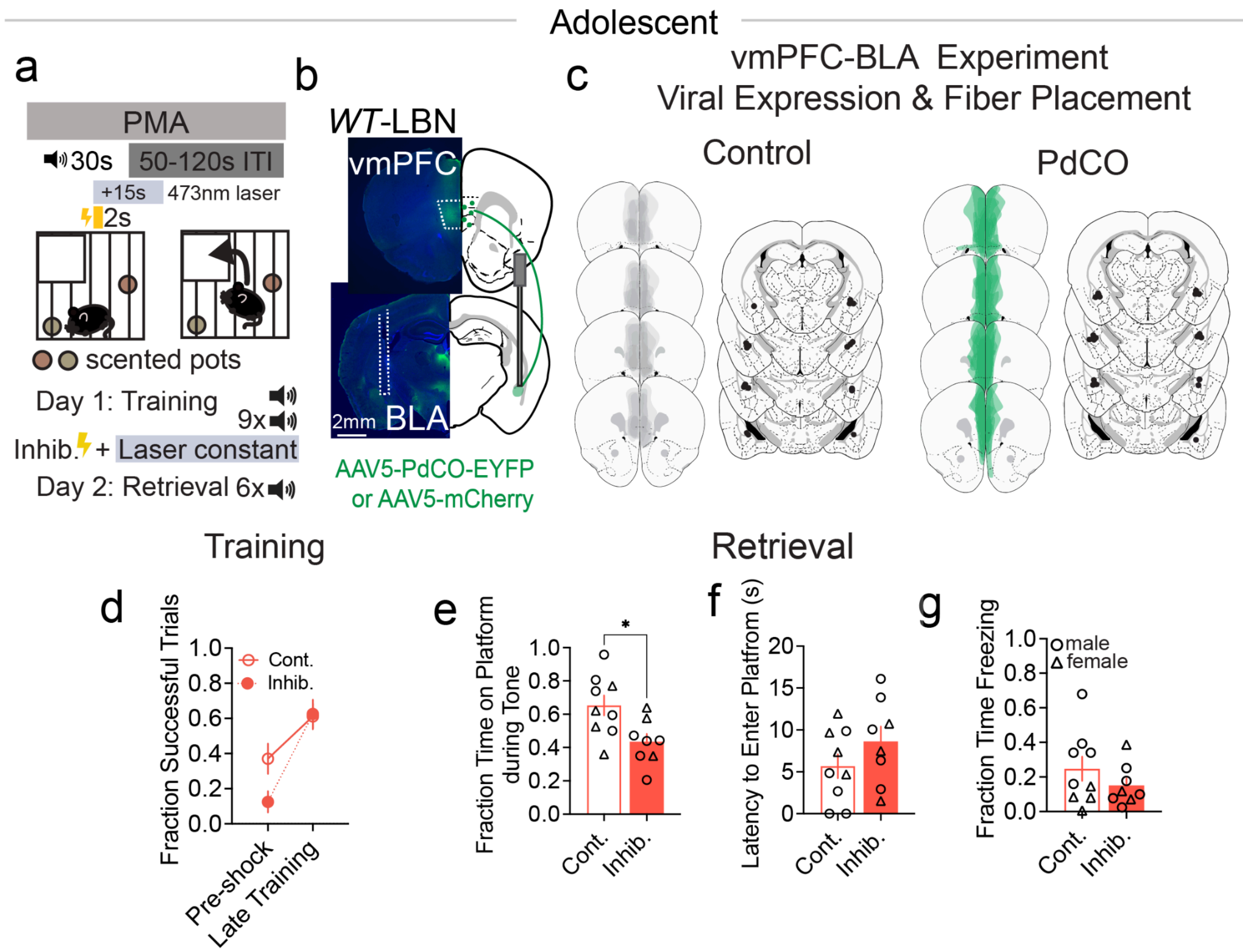

Extended Data Figure 7. Inhibition of vmPFC–BLA pathway reduces threat avoidance in LBN mice, Related to Figure 4. (**a**) Schematic representation of PMA paradigm with constant laser stimulation during the shock period and 13 seconds post shock during training. (**b**) Schematic and example images of AAV-PdCO expression and bilateral fiber optic implants in the BLA of wild-type LBN adolescent mice (Cont., n=9; Inhib., n=8). (**c**) AAV-PdCO viral expression in vmPFC in LBN mice and fiber implant placement above BLA. (**d**) Fraction of successful trials during PMA training averaged for the pre-shock baseline and the last four tones of the session (two-way ANOVA: trial effect $F_{1,15}$=41.52, p<0.0001; opsin effect $F_{1,15}$=1.55, p=0.232; interaction effect $F_{1,15}$=5.09, p=0.040). (**e-g**), Behavioral performance during PMA retrieval. (**e**) Fraction of time on the platform averaged across the session (unpaired t-test $F_{8,7}$=3.67, p=0.836). (**f**) Latency to enter the platform following tone onset (unpaired t-test $F_{7,8}$=1.36, p=0.213). (**g**) Fraction of time freezing during the tone (unpaired t-test $7_{8,7}$=3.18, p=0.270). Data are shown as mean +/- s.e.m.; *P<0.05. All data points represent biological replicates. Data are shown as mean +/- s.e.m. Triangles denote females, and circles denote males.

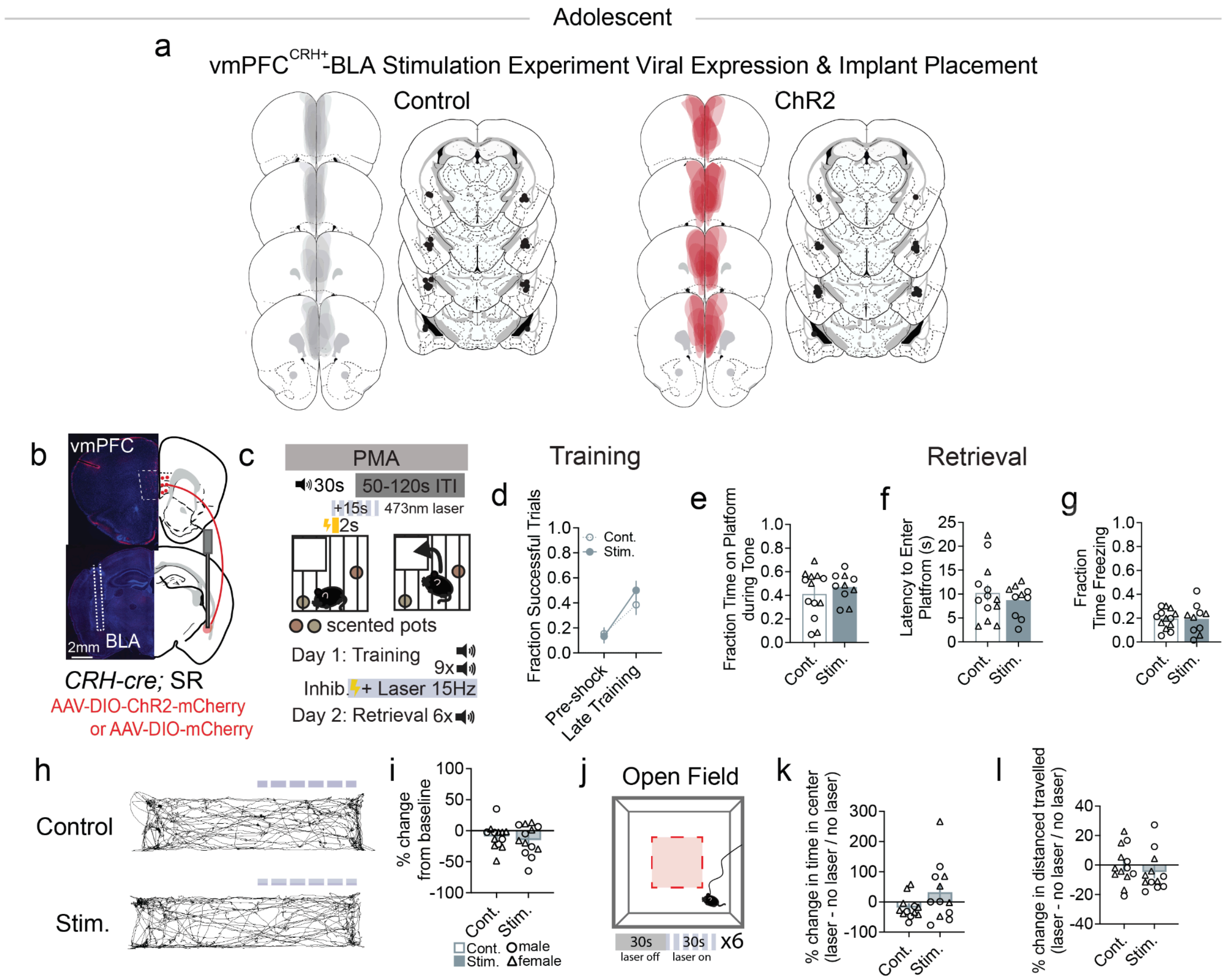

Extended Data Figure 8. Stimulation of the vmPFC^{CRH+}→ BLA pathway is not sufficient to drive increased avoidance in SR mice, Related to Figure 4. (**a**) AAV-ChR2 viral expression in mPFC of SR and LBN mice for stimulation experiments. (**b**) Schematic and example images of AAV-DIO-ChR2 expression and bilateral fiber optic implants in the BLA of CRH-cre mice. (**c**) Schematic representation of PMA with 15Hz laser stimulation during the shock period and 13 seconds post shock during training (Cont., n=13; Stim., n=10 mice). (**d**) Fraction of successful trials during PMA training averaged for the pre-shock baseline and the last four tones, only in SR mice (two-way ANOVA: trial effect $F_{1,21}$=23.77, p<0.0001; opsin effect $F_{1,21}$=0.45, p=0.510; interaction effect $F_{1,21}$=1.23, p=0.280). (**e-g**), Behavioral performance during PMA retrieval. (**e**) Fraction of time on the platform averaged across the session (unpaired t-test $F_{12,9}$=2.73, p=0.426). (**f**) Latency to enter the platform following tone onset (unpaired t-test $F_{12,9}$=3.18, p=0.481). (**g**) Fraction of time freezing during the tone (unpaired t-test $F_{12,9}$=2.33, p=0.976). (**h**) Representative mouse trajectory maps during RTPA with vmPFC^{CRH+}→BLA stimulation for control and ChR2-expressing mice in SR (cont., n=13; stim., n=13 mice). (**i**) Summary data of fraction of time spent on the laser-paired side of the chamber for stimulation experiments (unpaired t-test with Welch's correction: $F_{12,12}$=5.91, p=0.933). (**j**) Diagram of OF with 30 second 15Hz laser stimulation interleaved for 10 min (cont., n=13; stim., n=12 mice). Summary data of OF quantified as (**k**) percent change in time in center (unpaired t-test with Welch's correction: $F_{11,11}$=6.84, p=0.111) and (**l**) percent distance travelled as a function of laser stimulation (unpaired t-test: $F_{11,12}$=1.07, p=0.405). All data points represent biological replicates. Data are shown as mean +/- s.e.m.; Triangles denote females, and circles denote males.

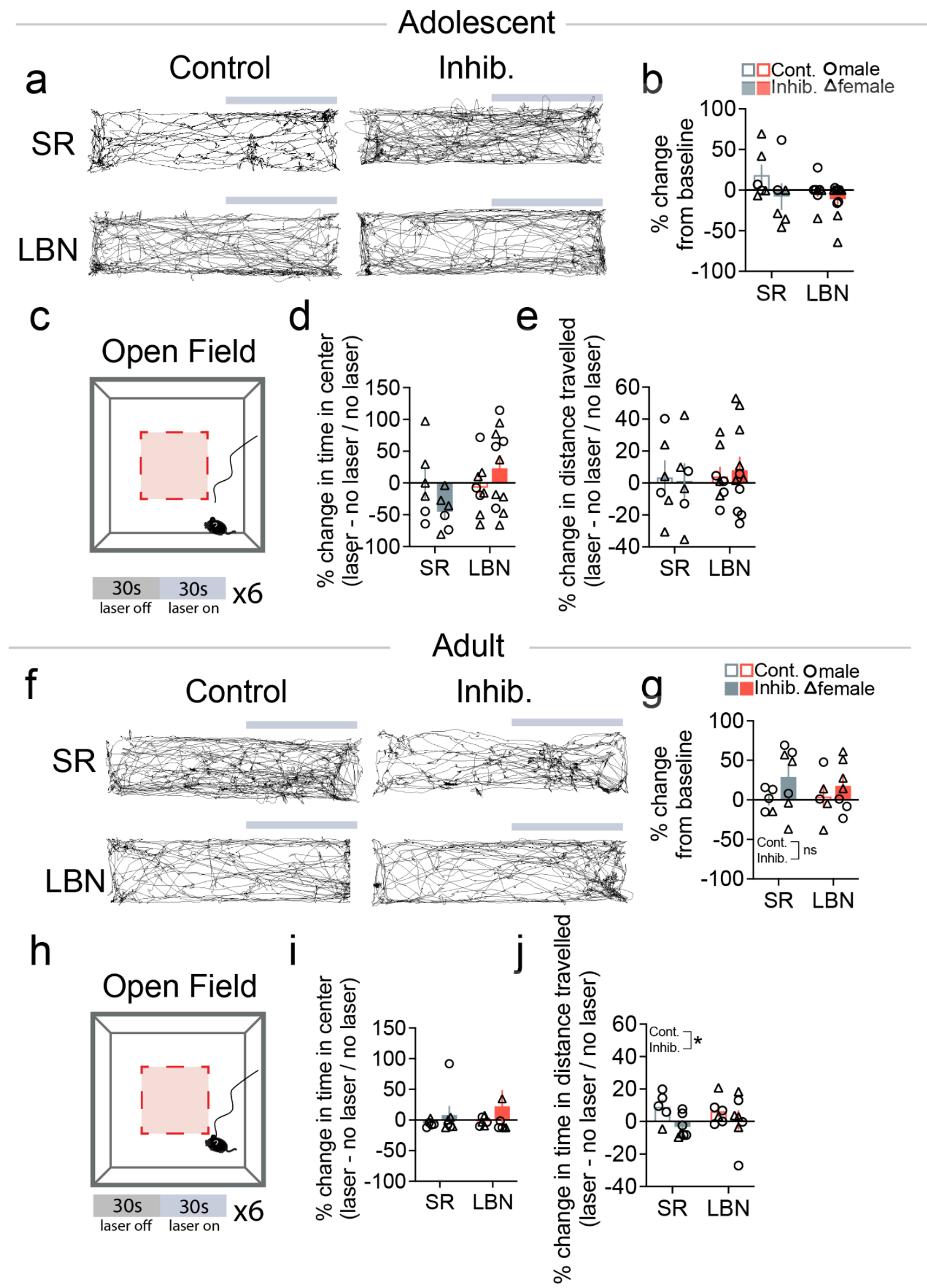

Extended Data Figure 9. Inhibition of vmPFC^{CRH+}→ BLA drives Real Time Place Preference only in adults, Related to Figure 4. (**a**) Representative mouse trajectory maps during RTPP in adolescents with vmPFC^{CRH+}→BLA inhibition for control and PdCO-expressing mice in SR and LBN groups (SR cont., n=6; SR inhib., n=6; LBN cont., n=8, LBN inhib., n=11 mice). (**b**) Summary data of fraction of time spent on the side of the laser-paired side of the chamber for inhibition experiments (two-way ANOVA: rearing effect $F_{1,27}$=1.43, p=0.242; opsin effect $F_{1,27}$=3.49, p=0.073; interaction effect $F_{1,27}$=0.391, p=0.761). (**c**) Diagram of OF with 30 second constant laser stimulation interleaved for 10 min in adolescents. Summary data of OF quantified as (**d**) percent change in time in center (two-way ANOVA: rearing effect $F_{1,27}$=2.50, p=0.126; opsin effect $F_{1,27}$=0.141, p=0.711; interaction effect $F_{1,27}$=3.79, p=0.062) and (**e**) percent distance travelled as a consequence of laser stimulation (two-way ANOVA: rearing effect $F_{1,27}$=0.166, p=0.687; opsin effect $F_{1,27}$=0.117, p=0.915; interaction effect $F_{1,27}$=0.122, p=0.730). (**f**) Representative mouse trajectory maps during RTPP in adults with vmPFC^{CRH+}→BLA inhibition for control and PdCO-expressing mice in SR and LBN groups (SR cont., n=5; SR inhib., n=7; LBN cont., n=5, LBN inhib., n=7 mice). (**g**) Summary data of fraction of time spent on the side of the laser-paired side of the chamber for inhibition experiments (two-way ANOVA: rearing effect $F_{1,20}$=0.078, p=0.883; opsin effect $F_{1,20}$=2.60, p=0.123; interaction effect $F_{1,20}$=0.033, p=0.572). (**h**) Diagram of OF with 30 second constant laser stimulation interleaved for 10 min in adults. Summary data of OF quantified as (**i**) percent change in time in center (two-way ANOVA: rearing effect $F_{1,21}$=0.265, p=0.612; opsin effect $F_{1,21}$=1.28, p=0.270; interaction effect $F_{1,21}$=0.103, p=0.751) and (**j**) percent distance travelled as a consequence of laser

stimulation (two-way ANOVA: rearing effect $F_{1,21}$=0.037, p=0.849; opsin effect $F_{1,21}$=4.479, p=0.0.046; interaction effect $F_{1,21}$=0.725, p=0.404). All data points represent biological replicates. Data are shown as mean +/- s.e.m.; Triangles denote females, and circles denote males.

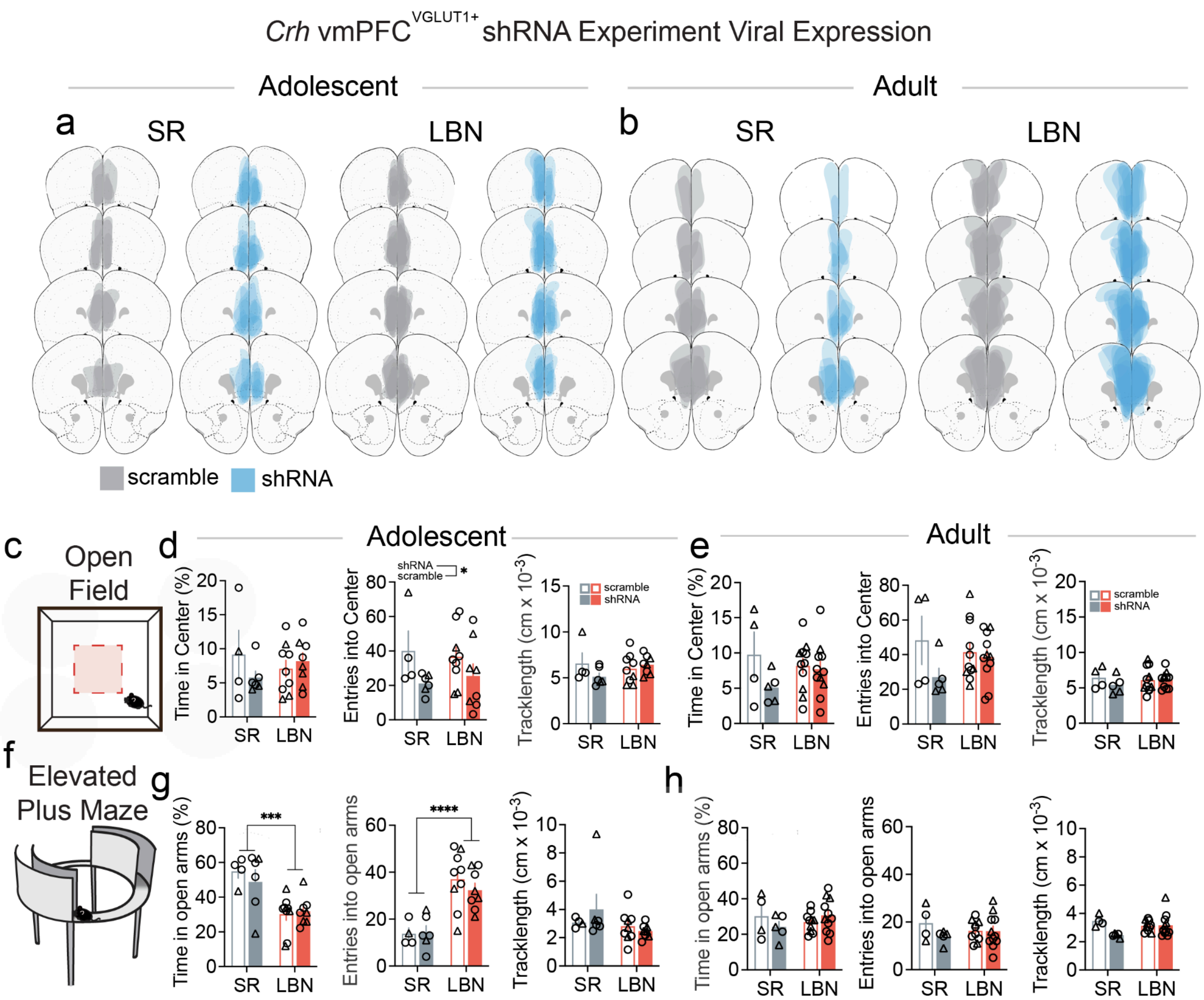

Extended Data Figure 10. ***Crh*** **knocdown in vmPFC$^{VGLUT1+}$ cells doesn't impact anxiety-like behavior, Related to Figure 5.** (**a**) AAV-shRNA viral expression in mPFC of VGLUT1-cre adolescent and (**b**) adult mice. (**c**) Schematic of OF in adolescent (SR cont., n=4; SR inhib., n=6; LBN cont., n=9, LBN inhib., n=8 mice) and adult (SR cont., n=4; SR inhib., n=5; LBN cont., n=11, LBN inhib., n=11 mice) mice. (**d**) Quantification of time in center (two-way ANOVA: knockdown effect $F_{1,23}$=0.494, p=0.489; rearing effect $F_{1,23}$=0.011, p=0.916; interaction effect $F_{1,23}$=1.86, p=0.186), entries into center (two-way ANOVA: knockdown effect $F_{1,23}$=4.65, p=0.0418; rearing effect $F_{1,23}$=0.0049, p=0.945; interaction effect $F_{1,23}$=0.30, p=0.588) and total tracklength travelled in cm in adolescent mice (two-way ANOVA: knockdown effect $F_{1,23}$=0.74, p=0.40; rearing effect $F_{1,23}$=0.409, p=0.529, interaction effect $F_{1,23}$=2.62, p=0.119) for vmPFC^{VGLUT1}+ Crh knockdown experiment. (**e**) Quantification of time in center (two-way ANOVA: knockdown effect $F_{1,16}$=1.82, p=0.106; rearing effect $F_{1,16}$=0.296, p=0.594; interaction effect $F_{1,16}$=1.52, p=0.235), entries into center (two-way ANOVA: knockdown effect $F_{1,16}$=1.71, p=0.210; rearing effect $F_{1,16}$=0.0004, p=0985; interaction effect $F_{1,16}$=1.63, p=0.221) and total tracklength travelled in cm in adolescent mice (two-way ANOVA: knockdown effect $F_{1,16}$=0.14, p=0.717; rearing effect $F_{1,16}$=0.078, p=0.784, interaction effect $F_{1,16}$=1.50, p=0.238) for vmPFC^{VGLUT1}+ Crh knockdown experiment. (**f**) Schematic of EZM. (**g**) Quantification of time in open arms (two-way ANOVA: knockdown effect $F_{1,23}$=0.226, p=0.639; rearing effect $F_{1,23}$=19.71, p=0.0002; interaction effect $F_{1,23}$=0.674, p=0.42), entries into open arms (two-way ANOVA: knockdown effect $F_{1,23}$=0.282, p=0.6004; rearing effect $F_{1,23}$=29.45, p<0.0001; interaction effect $F_{1,23}$=0.469, p=0.500) and total tracklength travelled (two-way ANOVA: knockdown effect $F_{1,23}$=0.351, p=0.559; rearing effect $F_{1,23}$=2.16, p=0.155; interaction effect $F_{1,23}$=1.43, p=0.244) for vmPFC$^{VGLUT1+}$ Crh knockdown experiment in adolescent mice. (**h**) Quantification of time in open arms (two-way ANOVA: knockdown effect $F_{1,27}$=2.74, p=0.109; rearing effect $F_{1,27}$=102, p=0.751; interaction effect $F_{1,27}$=1.78, p=0.193), entries into open arms (two-way ANOVA: knockdown effect $F_{1,27}$=3.15, p=0.087;

rearing effect $F_{1,27}$=0.12, p=0.723; interaction effect $F_{1,27}$=1.85, p=0.185) and total tracklength travelled (two-way ANOVA: knockdown effect $F_{1,27}$=0.72, p=0.402; rearing effect $F_{1,27}$=0.14, p=0.708; interaction effect $F_{1,27}$=0.89, p=0.354) for $vmPFC^{VGLUT1+}$ Crh knockdown experiment in adult mice. All data points represent biological replicates. Data are shown as mean +/- s.e.m.; Triangles denote females, and circles denote males.

Extended Data Table 1, Related to Figure 2.
Adolescent CRH+ cells counts normalized to region volume for SR and LBN mice, Related to Figure 2.

Region	SR 1	SR 3	SR 4	SR 5	SR 6	SR 8	LBN 1	LBN 2	LBN 3	LBN 4	LBN 5	LBN 6	LBN 7
Primary motor area'	9240.23	7779.58	10729.99	7983.33	8737.47	9078.82	6543.84	9348.72	8840.67	9078.82	7051.90	8192.37	8562.83
Secondary motor area'	9125.39	7285.18	10451.59	8068.44	8119.62	8613.61	6125.87	9265.58	8811.65	9169.90	7423.14	8010.59	8825.00
Primary somatosensory area'	6766.05	6132.42	8139.13	6555.70	7308.06	7337.74	5525.89	7732.63	7407.42	7832.00	5969.82	6396.97	7002.21
Supplemental somatosensory area'	9917.49	8538.47	10503.39	8820.71	9224.41	9871.05	8995.76	9806.74	10528.40	10564.13	8216.94	8567.05	9163.68
Gustatory areas'	6906.89	5479.11	7585.08	6960.43	6817.65	7852.79	6746.26	6853.34	7263.83	7906.33	6068.06	5746.81	6121.61
Visceral area'	10744.57	9491.51	11738.37	9693.15	10917.40	9865.99	9693.15	11133.44	11032.62	10154.05	8440.10	9160.25	10082.03
Dorsal auditory area'	11125.93	11151.69	10894.14	11151.69	11254.70	10404.81	11254.70	11563.76	11718.28	13109.03	9271.61	10147.26	11692.53
Primary auditory area'	11250.78	9835.02	10542.90	10151.31	11130.29	10618.21	10663.39	10994.74	10844.13	11115.23	9654.29	9443.43	11190.54
Posterior auditory area'	11639.13	10254.74	12510.78	11536.58	11946.77	11690.40	10767.47	11331.48	10767.47	11792.95	9998.37	10459.83	11741.67
Ventral auditory area'	9166.62	8398.37	8607.89	8276.15	9026.94	9219.00	7717.42	9149.16	8276.15	9672.96	7699.96	7769.80	9009.48
Anterolateral visual area'	10941.49	11983.54	11542.67	10741.10	11382.36	11662.91	11702.99	11302.20	13466.45	11863.30	10139.92	11101.81	11983.54
Anteromedial visual area'	5788.88	6852.14	8309.20	5198.17	5119.41	6930.90	3268.55	6930.90	6064.54	6615.86	5670.74	6418.96	10160.07
Lateral visual area'	12582.47	12884.45	13563.91	11273.90	12381.16	13639.40	11525.55	12733.46	14016.88	13337.42	12079.18	11777.20	12808.96
Primary visual area'	13222.84	12197.94	14033.88	12218.11	13828.09	14025.81	11665.31	13037.23	13045.30	13436.69	11826.71	12452.14	13634.41
Posterolateral visual area'	12286.86	9702.02	10658.06	11684.91	10551.83	10905.92	10799.69	10233.15	10693.47	12145.23	10622.65	10091.52	11153.78
posteromedial visual area'	8729.89	8499.39	13109.23	7490.99	8528.21	8557.02	6338.53	7980.79	9507.80	8672.26	8096.03	8067.22	8701.07
dmPFC'	12624.01	9509.11	14461.75	12210.95	10874.90	12501.80	10697.82	14499.74	10942.24	11507.05	10293.21	11696.79	13037.06
vmPFC'	20549.44	11677.10	17636.95	15371.68	17097.66	14832.19	9681.37	13753.63	12701.79	14913.20	13753.63	12701.79	12108.44
Orbital area, lateral part'	6364.02	5071.33	7408.12	5170.77	5991.13	5916.55	4822.74	6202.44	6910.93	8240.91	4586.57	5058.90	5580.95
Orbital area, medial part'	5924.30	3748.02	6770.62	4014.01	5779.21	5102.15	4860.34	5295.60	6601.36	10857.18	4352.54	4932.88	5174.69
Orbital area, ventrolateral part'	4850.84	4338.15	7749.52	4752.25	6526.95	6428.35	4771.97	6310.04	9050.96	9977.75	4771.97	5955.10	5895.94
Agranular insular area, dorsal part'	7910.62	6567.75	9099.15	7115.70	7216.03	8188.46	6891.89	8443.14	8489.45	9284.37	6606.34	6961.35	7254.62
Agranular insular area,	9410.15	7914.95	10356.65	8902.61	9958.85	9286.69	8532.24	10000.00	10027.43	10507.54	8010.97	8230.45	9423.87

posterior part'													
Agranular insular area, ventral part'	9072.01	8442.01	10926.02	8964.01	9738.01	10926.02	8442.01	9828.01	10350.02	10350.02	7722.01	8856.01	9450.01
Retrosplenial area'	10740.43	10403.14	13110.68	10146.31	10192.73	11845.10	6857.04	11876.05	10028.73	10721.86	9750.24	11708.95	12640.35
Rostrolateral visual area'	5436.08	5198.59	5436.08	5172.20	5647.19	5568.03	4248.59	5911.08	6069.41	6386.08	4380.53	4301.37	5726.36
Temporal association areas'	8204.37	6845.36	6905.76	6956.10	7640.63	7348.70	7640.63	6976.23	7399.03	7248.03	6633.96	6784.96	7862.10
Perirhinal area'	7438.64	5406.62	6168.63	5842.05	5551.76	5805.77	5624.34	5551.76	5950.91	5588.05	5624.34	5188.90	6313.77
Ectorhinal area'	7811.94	7882.00	7846.97	7303.99	8284.86	7776.91	7391.57	8337.41	7444.11	7846.97	6708.46	7409.08	8670.20
Accessory olfactory bulb'	1394.55	1704.45	1756.10	1342.90	1033.00	2375.90	1549.50	2117.65	1394.55	7437.60	1652.80	1394.55	2427.55
Anterior olfactory nucleus'	6723.86	4827.39	8340.18	6048.60	6084.52	6228.19	4489.76	7176.43	6925.01	8835.85	4820.21	5653.51	5725.34
Taenia tecta'	15707.37	8155.75	11352.60	10421.24	13391.54	11503.64	9212.98	11075.71	11000.20	14750.83	9162.63	9313.67	8432.64
Piriform area'	9330.09	8361.02	10392.12	8397.64	9631.51	9448.41	8121.57	9493.48	10147.03	10163.94	7521.54	8318.77	8848.37
Nucleus of the lateral olfactory tract'	4338.75	4751.97	7541.17	4751.97	6198.22	5165.18	2995.81	5681.70	5888.31	5578.40	5578.40	3822.24	4855.27
Cortical amygdalar area, anterior part'	7289.93	6682.44	8059.42	5831.94	7370.93	6479.94	4292.96	8383.42	8221.42	8585.92	5426.95	6317.94	6925.43
Cortical amygdalar area, posterior part'	10248.91	10860.95	11817.96	10438.08	11651.04	11306.07	8802.27	10961.10	11996.01	12296.46	9592.35	10538.24	11940.37
Piriform-amygdalar area'	8318.71	9251.90	9651.84	8132.07	9705.16	9731.82	8931.95	9545.19	10718.34	10558.36	6825.61	8665.32	9785.15
Postpiriform transition area'	12218.95	12007.55	12916.57	12366.93	12134.39	13508.49	11753.87	12366.93	12388.07	12726.31	10781.42	12430.35	14502.07
Field CA1'	10774.30	10662.86	11829.77	9928.62	10810.36	11524.93	9102.60	10970.98	11000.48	11688.83	9548.39	10423.57	11987.11
Field CA2'	10628.42	9662.20	11456.61	9869.25	10076.30	10904.48	8903.03	12215.78	10283.34	9800.23	8419.92	7246.65	11939.72
Field CA3'	9858.78	10175.79	11660.42	8997.59	10233.90	10577.32	7703.17	10239.19	11147.93	11015.84	8765.12	10001.44	10778.09
Dentate gyrus'	10037.52	10143.34	12222.80	9915.82	10302.08	11005.82	7640.58	10937.03	9809.99	10741.25	9534.85	10354.99	11905.33
Entorhinal area, lateral part'	6884.85	5646.04	5751.96	5996.04	6175.64	6207.88	6014.46	6166.43	6051.30	5968.41	5747.36	5586.17	6553.28
Entorhinal area, medial part, dorsal zone'	9554.02	8450.92	9063.75	9131.16	8935.06	9566.27	8916.67	9265.99	8463.18	9302.76	9039.24	8873.77	10019.77
Parasubiculum'	13256.51	12268.23	12574.94	13426.90	13835.84	14790.04	12404.55	13017.96	10768.78	14347.02	12643.10	12540.86	12915.72
Postsubiculum'	12999.05	12503.17	13778.28	13424.09	13388.67	14451.26	12007.30	12821.95	13069.89	14380.42	12467.75	12821.95	14415.84
Presubiculum'	13790.56	13212.95	16101.03	12527.03	13682.26	15126.30	12851.94	13465.66	13357.35	15306.80	13140.75	13429.55	14368.18

Subiculum'	12684.48	12117.49	13656.48	12263.29	12538.69	13850.87	11550.50	12538.69	12311.89	13899.47	12311.89	12344.29	12830.28
Cortical subplate'	8446.90	8527.35	9734.05	7079.31	8688.24	9653.60	7320.65	9492.71	9412.26	9573.15	9090.47	6596.63	9010.03
Claustrum'	9893.82	9838.85	12367.27	9399.12	10828.23	9728.92	8464.71	9893.82	11432.85	11927.54	9179.26	7255.46	8189.88
Endopiriform nucleus'	9255.86	8743.46	10237.04	8525.42	10269.75	10171.63	9005.11	10324.26	10651.32	11163.72	8143.84	8787.07	9462.99
Lateral amygdalar nucleus'	9205.91	10277.83	10593.10	10025.61	9931.03	10057.14	10530.05	10246.30	11507.39	13272.90	9426.60	9836.45	10719.21
Basolateral amygdalar nucleus, anterior part'	8146.43	8146.43	10819.47	8178.25	9705.70	9228.37	8019.14	9164.73	10469.43	9387.48	8337.36	7955.49	9260.19
Basolateral amygdalar nucleus, posterior part'	10993.48	9940.21	10368.10	7833.68	10598.51	11289.71	10763.08	11256.80	13659.56	12935.44	8393.23	8722.37	11289.71
Basolateral amygdalar nucleus, ventral part'	8856.46	9626.59	11744.44	8471.40	11616.09	10140.01	11359.38	10011.65	12193.68	12065.33	8471.40	8984.82	8920.64
Basomedial amygdalar nucleus, anterior part'	7033.45	7267.90	8322.92	7736.80	7814.95	8166.62	5861.21	7932.17	8166.62	8635.51	6017.51	6486.40	7932.17
Basomedial amygdalar nucleus, posterior part'	8904.53	11130.66	12066.28	10001.46	11130.66	11259.71	8936.79	11066.13	12195.33	12163.07	10162.77	10001.46	11453.29
Posterior amygdalar nucleus'	10363.87	8701.21	12248.21	8978.32	11610.86	11278.33	7537.36	10086.76	11278.33	12442.18	8867.48	9116.88	12109.65
Caudoputamen'	6637.72	5812.19	8237.85	6330.83	7746.01	7050.48	4831.21	7825.08	6868.22	6948.63	5737.14	6237.02	7082.65
Nucleus accumbens'	11257.00	9212.15	14293.29	10361.95	10031.47	10788.82	6382.41	12854.33	9611.48	9184.61	9336.08	10072.78	11973.04
Olfactory tubercle'	9830.38	7612.24	11099.08	8309.61	10141.25	9107.80	5755.39	10149.65	8864.14	8402.03	8133.17	8402.03	9813.57
Lateral septal complex'	10493.32	9297.39	14659.78	11361.33	9509.57	11390.27	7175.58	13994.31	10657.28	8593.33	10242.56	12402.95	15739.98
Striatum-like amygdalar nuclei'	4809.26	5771.11	7969.63	5816.91	6549.75	6320.74	3755.80	6824.57	5862.72	6229.14	5725.31	6045.93	7878.02
Central amygdalar nucleus'	7384.42	6836.46	8193.31	7593.17	7828.01	7880.19	5949.29	9628.45	9002.21	7932.38	5897.10	7332.23	7958.47
Medial amygdalar nucleus'	6188.12	5940.59	9947.40	6280.94	6234.53	6296.41	3357.05	7518.56	6590.35	7534.03	5878.71	7193.69	8493.19
Globus pallidus, external segment'	6052.58	5483.53	8225.30	6932.02	7216.54	6466.43	3931.59	7811.45	5897.39	7811.45	5612.86	6569.90	7992.51
Globus pallidus, internal segment'	5055.64	7058.82	10397.46	5914.15	6486.49	7631.16	2480.13	8489.67	5914.15	6486.49	8489.67	8489.67	10302.07
Substantia innominata'	8026.51	7428.67	10749.99	7860.45	7052.26	9011.83	4494.85	9997.16	7738.66	7971.16	7926.87	8347.57	10528.57
Magnocellular nucleus'	6638.88	6551.53	11618.04	8036.54	7250.36	9696.26	3494.15	10307.74	6900.94	6289.47	9172.14	9521.55	9870.97

Medial septal nucleus'	14986.11	15913.08	16994.55	20934.20	14136.38	23251.64	6643.33	23792.38	14522.62	12436.92	8265.53	19003.00	13132.16
Diagonal band nucleus'	12389.65	9752.50	13683.35	12439.41	12091.11	13235.53	5970.92	15225.84	12389.65	9205.16	9105.65	10250.08	11941.84
Triangular nucleus of septum'	10514.49	11111.90	18997.77	12306.73	9917.08	12545.70	6452.07	17444.49	8961.21	8363.80	11350.87	14815.87	15413.29
Bed nuclei of the stria terminalis'	11753.89	6309.99	7943.16	5518.14	6804.89	7720.45	4874.77	7349.28	9205.15	7027.59	5419.16	7349.28	7126.57
Ventral group of the dorsal thalamus'	7220.74	7085.40	9959.36	6894.33	6098.22	8279.57	3725.80	8757.23	6233.56	7690.44	6902.29	8104.42	9959.36
Subparafascicular nucleus'	6778.20	9037.60	12991.55	6401.63	6401.63	9225.88	5648.50	5271.93	7343.05	8661.03	9602.45	11108.72	12238.42
Subparafascicular area'	11001.80	8975.15	13317.97	8975.15	10133.24	11870.36	6079.94	11001.80	8396.11	9264.67	15634.14	15344.62	9554.20
Peripeduncular nucleus'	12508.89	14295.87	15487.20	11317.57	14295.87	10721.91	11913.23	12508.89	10721.91	13700.21	7147.94	11317.57	10721.91
Geniculate group, dorsal thalamus'	11155.38	10885.93	13957.70	10508.69	12556.54	14281.04	8945.86	11586.51	12664.32	10993.71	9484.77	12340.98	9808.11
Lateral group of the dorsal thalamus'	7040.13	8077.50	11003.12	7378.15	7261.59	9382.95	4301.01	9452.89	6748.73	7844.38	6923.57	7925.97	10991.46
Anterior group of the dorsal thalamus'	8987.17	7358.83	10302.37	7233.58	7812.89	9378.60	3742.05	8987.17	7061.35	7108.32	7233.58	8783.63	8799.29
Medial group of the dorsal thalamus'	11516.31	11441.77	12541.22	10901.36	9969.62	11702.66	5720.88	12858.01	10137.33	10211.87	9522.38	12727.57	15709.14
Midline group of the dorsal thalamus'	10925.79	12425.41	14567.72	12517.22	9273.15	13129.31	5845.45	15149.21	9671.01	7987.76	11231.84	13527.17	16342.78
Intralaminar nuclei of the dorsal thalamus'	10061.56	11081.14	12556.83	10410.37	9739.59	11751.91	5688.14	12503.17	8344.39	9551.78	8129.74	11322.61	15213.08
Reticular nucleus of the thalamus'	7318.36	7237.35	9262.72	7723.44	6211.16	8560.59	4023.75	8236.53	7183.34	7912.47	7696.43	7777.45	8749.63
Intergeniculate leaflet of the lateral geniculate complex'	10534.59	11705.10	10534.59	6437.81	16972.40	9364.08	8193.57	9364.08	11705.10	14046.12	9949.34	7608.32	9364.08
Ventral part of the lateral geniculate complex'	10465.58	9967.22	12957.39	9468.86	11661.65	10066.89	7276.07	10465.58	13455.75	11860.99	9070.17	9269.52	10864.27
Subgeniculate nucleus'	1645.52	6582.07	14809.66	9873.11	14809.66	14809.66	3291.04	18100.70	8227.59	14809.66	3291.04	4936.55	4936.55
Medial habenula'	11474.68	14089.16	13072.42	8714.94	9876.94	13362.91	7262.45	11038.93	9876.94	8569.69	10312.68	16558.39	18446.63

Lateral habenula'	12138.28	12579.67	13352.11	11696.89	11586.54	12579.67	7393.32	13683.15	9931.32	10593.41	9379.58	12690.02	17766.03
Periventricular zone'	12101.92	10968.63	15178.00	13356.64	9592.49	13963.76	6192.62	15420.85	9471.07	9228.22	11413.85	12709.04	14206.61
Periventricular region'	9783.14	10633.18	13322.38	10540.45	9814.05	11823.23	5857.52	13322.38	9659.50	8794.01	9134.02	12704.18	13507.85
Anterior hypothalamic nucleus'	8866.36	7549.58	11104.90	8646.90	7681.25	8954.15	3204.18	9480.86	8515.22	7374.00	7330.11	8515.22	10973.22
Mammillary body'	12198.38	11548.76	15121.66	11296.13	10069.07	13461.52	7687.14	13858.51	8986.38	11909.66	9960.80	13750.24	12667.54
Medial preoptic nucleus'	8212.07	7596.16	12318.11	9922.92	6911.83	7596.16	4585.07	10196.65	8554.24	5474.71	8691.11	11496.90	17450.65
Dorsal premammillary nucleus'	9984.37	13214.60	11746.31	8516.08	9690.71	10278.03	7928.76	7928.76	10278.03	6460.47	9397.05	11452.66	12920.95
Ventral premammillary nucleus'	9210.55	10315.82	11421.08	10500.03	10315.82	10500.03	7736.86	9394.76	8473.71	11052.66	8473.71	11605.29	15105.30
Paraventricular hypothalamic nucleus, descending division'	12802.72	6668.08	13336.16	7201.53	8535.15	10402.21	4801.02	9602.04	4801.02	8535.15	9868.76	15203.23	12535.99
Ventromedial hypothalamic nucleus'	11845.14	11119.93	15108.60	12026.45	11361.67	12026.45	5801.70	13476.87	10032.11	11724.27	10152.98	15652.51	15833.81
Posterior hypothalamic nucleus'	11445.63	11047.02	13438.65	8826.23	10022.04	13780.31	8199.85	12869.21	9395.66	10249.81	9110.95	11502.57	14235.85
Hypothalamic lateral zone'	8310.76	7925.26	9682.44	7862.50	6526.68	9368.66	3612.98	9296.93	6867.36	7772.85	7037.70	8463.17	10570.00
Zona incerta'	7495.29	7151.66	9943.61	6357.04	6464.42	7946.29	3285.90	8268.44	6936.90	7430.86	7259.05	7989.25	9148.98
Median eminence'	12830.13	12830.13	10157.19	11760.95	8018.83	12830.13	8018.83	12295.54	7484.24	10691.77	14433.89	14968.48	12295.54
Superior colliculus, sensory related'	13192.88	11479.05	13674.89	12300.26	12211.00	13960.53	10050.87	13139.32	11050.60	14014.08	11086.30	12228.85	12710.86
Inferior colliculus'	11350.75	11166.76	12482.99	11633.81	11796.57	12801.43	11463.97	12107.94	11159.68	11902.72	11046.46	11860.26	12801.43
Substantia nigra, reticular part'	10178.15	9624.24	12647.68	9324.20	10178.15	10016.59	7639.38	9601.16	10062.75	10270.47	8862.61	10016.59	10755.14
Ventral tegmental area'	10196.46	8676.49	13173.06	9436.47	6966.52	10196.46	7093.19	10893.11	8676.49	10513.12	8993.15	10766.44	12223.08
Superior colliculus, motor related'	11918.18	10826.82	13804.56	10862.96	11744.72	13060.13	9439.14	11491.76	11549.58	12597.57	10364.26	11527.89	12221.74
Periaqueductal gray'	13603.70	12811.00	13671.89	13586.65	13970.21	15172.04	9947.06	14294.11	11881.93	14413.44	12112.06	13884.98	14396.39
Precommissural nucleus'	10591.83	12945.57	13416.32	7061.22	8002.72	12004.08	7531.97	10356.46	10591.83	11062.58	8002.72	12710.20	15299.31
Anterior pretectal nucleus'	8924.21	8381.52	11456.75	6904.20	6602.71	8411.67	4763.60	7145.40	6843.90	8713.16	8321.22	8411.67	9255.85
Medial pretectal area'	11653.62	8324.02	10821.22	11653.62	5826.81	14983.23	5826.81	10821.22	9156.42	9156.42	9156.42	11653.62	9988.82

Substantia nigra, compact part'	9662.49	8309.74	13140.98	6183.99	7246.86	9082.74	5700.87	10338.86	8986.11	9179.36	7150.24	8599.61	9469.24
Pedunculopontine nucleus'	12689.73	11595.78	14658.82	12821.00	13827.43	15577.73	13083.54	14790.09	14046.21	14615.06	13302.33	14790.09	14046.21
Interfascicular nucleus raphe'	24062.99	18047.24	21749.24	19898.24	16658.99	19435.49	12031.49	15733.49	11568.74	23600.24	15270.74	22674.74	17584.49
Interpeduncular nucleus'	23017.46	23017.46	17263.10	17263.10	23017.46	25894.65	5754.37	14385.91	14385.91	25894.65	23017.46	17263.10	8631.55
Rostral linear nucleus raphe'	25873.98	28597.56	20426.83	17022.36	14298.78	29959.35	21107.72	26554.88	10894.31	35406.50	12936.99	24512.20	14979.67
Central linear nucleus raphe'	15867.77	22314.05	20330.58	14876.03	22809.92	23305.79	18842.98	21322.31	12892.56	25785.12	23305.79	16859.50	15867.77
Dorsal nucleus raphe'	22130.47	8694.11	16334.40	10538.32	16597.85	9484.49	17124.77	13963.27	21867.01	26609.26	20549.72	11592.15	7376.82
